# Sustainable β-carotene production by engineered *S. cerevisiae* using sucrose and agricultural by-products

**DOI:** 10.1186/s40643-025-00936-y

**Published:** 2025-09-13

**Authors:** Suriyaporn Bubphasawan, Kitisak Sansatchanon, Peerada Promdonkoy, Akaraphol Watcharawipas, Sutipa Tanapongpipat, Peerapat Khamwachirapithak, Weerawat Runguphan, Kanokarn Kocharin

**Affiliations:** 1 Industrial Bioprocess Innovation Group, The Eastern Economic Corridor of Innovation, National Science and Technology Development Agency (NSTDA), 333 EECi Headquarters Wang Chan, Rayong, 21210 Thailand; 2 National Center for Genetic Engineering and Biotechnology, 113 Thailand Science Park, Paholyothin Road, Klong 1, Klong Luang, 12120 Pathumthani Thailand; 3 Department of Microbiology, Faculty of Science, Mahidol University, 272 Rama VI Road, Ratchathewi, Bangkok, 10400 Thailand; 4 Department of Biotechnology, Faculty of Science and Technology, Thammasat University, Rangsit Campus, Phahonyothin Road, Khlong Luang, 12120 Pathumthani Thailand

**Keywords:** Carotenoids, Β-carotene, Yeast, Agricultural wastes, Bioprocess development

## Abstract

β-carotene, a carotenoid precursor to vitamin A, is widely employed in the food, pharmaceutical, and nutraceutical sectors. In this study, we present an economically sustainable strategy for β-carotene biosynthesis in *Saccharomyces cerevisiae* by engineering the yeast to utilize sucrose and agricultural by-products as alternative carbon and nitrogen sources. Specifically, the deletion of the *GAL80* gene facilitated effective β-carotene synthesis directly from sucrose, circumventing the costly requirement for galactose induction. Using this engineered yeast strain, we achieved β-carotene titers of up to 23.30 ± 4.22 mg/L and content levels of 2.29 ± 0.16 mg/g dry cell weight (DCW). To further improve the economic viability and environmental sustainability, we evaluated the use of agricultural by-products—molasses as a carbon source and fish meal as a nitrogen source—in a fed-batch fermentation process, highlighting the potential of these substrates to replace refined feedstocks while achieving competitive β-carotene production levels. This approach yielded substantial β-carotene titers of 17.02 ± 0.40 mg/L and content levels of 2.90 ± 0.21 mg/g DCW. It also significantly reduced medium costs by up to 73% compared to conventional yeast extract and peptone-based media, demonstrating the practical potential of these low-cost, sustainable substrates for industrial applications. This study uniquely highlights the successful application of unrefined agricultural by-products, addressing key challenges in cost and sustainability. These findings represent an important advancement toward developing economically competitive and environmentally responsible microbial platforms for the production of β-carotene and other high-value biochemicals.

**Supplementary Information:**

The online version contains supplementary material available at 10.1186/s40643-025-00936-y.

## Graphical abstract

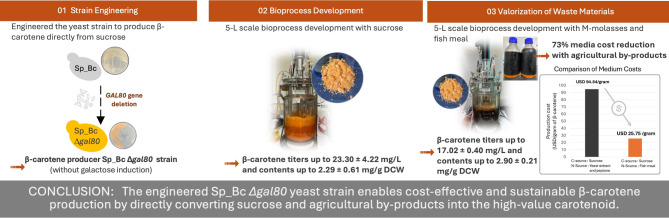

## Introduction

β-carotene, an orange-red pigment in plants, plays an essential role as a vitamin A precursor, offering wide-ranging benefits for vision, immunity, and skin health (Wang et al. 2021; Watcharawipas and Runguphan 2023). Its significant nutritional value has led to increasing commercial interest, boosting demand across global markets (Bogacz-Radomska et al. 2020; Singh and Sambyal 2022). Traditional extraction from sources such as carrots and pumpkins faces scalability challenges, including limited yields and land constraints, which has necessitated the exploration of microbial synthesis as a scalable alternative (Jing et al. 2022; Guo et al. 2023).

The yeast *Saccharomyces cerevisiae*, commonly known as baker’s yeast, stands out as a favorable host for metabolic engineering due to its extensively documented metabolic pathways and genetic tractability (Baptista et al. 2021). Recent advances in metabolic engineering and fermentation optimization have enabled engineered *S. cerevisiae* strains to achieve β-carotene titers ranging from several hundred milligrams per liter to over 2 g/L in fed-batch bioreactor processes. For example, López et al. (2019) reported high β-carotene production by overexpressing carotenogenic genes *CrtE*, *CrtYB*, and *CrtI* from the red yeast *Xanthophyllomyces dendrorhous* as well as overexpressing the endogenous gene *tHMG1*, resulting in a titer and content of 740 mg/L and 6.9 mg/g DCW, respectively, in glucose-fed batch cultures (López et al. 2019). Sun et al. (2020) further improved the titer and content to 772.8 mg/L and 11.4 mg/g DCW by applying adaptive laboratory evolution and multiple genetic modifications that enabled β-carotene production from xylose (Sun et al. 2020). Fathi et al. (2021) explored the synergistic effect of lipid metabolism engineering and carotenogenic gene overexpression, reaching a titer and content of 477.9 mg/L and 46.5 mg/g DCW, respectively, in a glucose and olive oil medium (Fathi et al. 2021). Most recently, Lin et al. (2025) achieved the highest reported titer to date (2.09 g/L in a 5-L fed-batch bioreactor) by integrating multi-layer pathway optimization, enhancing precursor supply, remodeling lipid droplets, and applying atmospheric and room temperature plasma (ARTP) mutagenesis to generate a hyper-producing strain (Lin et al. 2025). Despite these impressive advancements, reliance on refined substrates significantly increases production costs and poses limitations for commercial scalability. Therefore, exploring alternative, sustainable, low-cost, and readily available agricultural wastes as substrates for β-carotene production in yeast is critical for developing economically viable and environmentally sustainable industrial bioprocesses.

Previous studies have demonstrated the utlility of novel *crt* gene variants from the red yeast *Sporidiobolus pararoseus* to improve lycopene and β-carotene yields (Watcharawipas et al. 2021). The *Sp*CrtI enzyme exhibited five-fold higher enzymatic activity compared to its counterparts from *X. dendrorhous*, leading to a seven-fold increase in β-carotene content. However, such initial work relied heavily on galactose for induction, significantly increasing overall production costs and limiting commercial feasibility (Kim et al. 2022).

In the current study, this limitation was addressed by engineering *S. cerevisiae* to bypass the requirement for galactose, enabling direct β-carotene synthesis from sucrose. Furthermore, the practical application of sustainable agricultural by-products, specifically molasses as an alternative carbon source and fish meal as an alternative nitrogen source, was evaluated to substantially reduce production costs. These inexpensive substrates align closely with circular economy principles, facilitating the conversion of waste streams into valuable bioproducts and significantly reducing environmental impact (Duque-Acevedo et al. 2020). Collectively, this approach integrates innovative strain design and strategic bioprocess optimization, marking an important advancement toward economically viable and environmentally sustainable microbial β-carotene production platforms.

## Materials and methods

### Strain, media, transformation, and primers

The parental *Saccharomyces cerevisiae* strain used in this study was Sp_Bc, derived from *S. cerevisiae* CEN.PK2-1 C and constructed in our previous work (Watcharawipas et al. 2021). This strain contains multiple chromosomally integrated copies of mevalonate and carotenoid biosynthetic genes, each under the control of *GAL1* and *GAL10* promoters, to enable high-level β-carotene biosynthesis upon galactose induction. The complete genotype is: CEN.PK2-1 C, *416d::HIS3 T*_*HMG1*_*-tHMGR-P*_*GAL1*_*-P*_*GAL10*_*-ERG12-T*_*ERG12*_, *308a::LEU2 T*_*HMG1*_*-tHMGR-P*_*GAL1*_*-P*_*GAL10*_*-ERG8-T*_*ERG8*_, *720a::TRP1 T*_*HMG1*_*-tHMGR-P*_*GAL1*_*-P*_*GAL10*_*-ERG19-T*_*ERG19*_, *SAP155c::loxP T*_*ERG13*_*-ERG13-P*_*GAL1*_*-P*_*GAL10*_*-IDI1-T*_*IDI1*_, *YPRCd15c::loxP T*_*ERG10*_*-ERG10-P*_*GAL1*_*-P*_*GAL10*_*-ERG20-T*_*ERG20*_, *1021b::T*_*PRM9*_*-SpCrtE(opt)-P*_*GAL1*_*-P*_*GAL10*_*-SpCrtYB(opt)-T*_*GAL10*_*-P*_*GAL7*_*-SpCrtI(opt)-T*_*CPS1*_. Here, *tHMGR* represents the truncated HMG-CoA reductase, and *SpCrtE(opt)*, *SpCrtYB(opt)*, and *SpCrtI(opt)* are genes from *Sporidiobolus pararoseus* with codons optimized for gene expression in *S. cerevisiae*. This design enhances precursor supply via the mevalonate pathway and directs metabolic flux toward β-carotene biosynthesis. For cloning and plasmid construction, *Escherichia coli* DH5α, obtained from Invitrogen, was utilized. *S. cerevisiae* was typically grown in either yeast extract-peptone-dextrose (YPD) medium (1% yeast extract, 2% peptone, and 2% glucose) or a synthetic complete (SC) medium (0.67% yeast nitrogen base without amino acids, 2% glucose, and suitable amino acid and nucleobase supplements). The pH of the SC medium was adjusted to 6.0 using 1 N potassium hydroxide. For galactose induction, the SC medium was supplemented with glucose or sucrose alongside galactose. *E. coli* DH5α was grown in Luria-Bertani (LB) medium (0.5% yeast extract, 1% peptone, and 0.5% sodium chloride) supplemented with 100 µg/mL ampicillin when required. DNA introduction into yeast cells was achieved using the LiAc/SS carrier DNA/PEG procedure, as described by Gietz and Schiestl in 2007 (Gietz and Schiestl 2007). Selection of yeast transformants was based on either their nutritional requirements (auxotrophy) or antibiotic resistance, utilizing 200 µg/mL G418 or hygromycin B for selection.

In fermentations conducted on a 5-L scale, sucrose and galactose, sourced from Mitr Phol ($0.70/kg) and Difco ($252.45/kg) respectively, served as carbon sources. M-molasses, used as an alternative carbon source, was also obtained from Mitr Phol at a cost of $0.56/kg. Traditional nitrogen sources, yeast extract and peptone, were procured from Himedia at costs of $62.27/kg and $71.81/kg, respectively. Alternatively, fish meal was used as a cost-effective nitrogen source, purchased from High-Quality Fish Meal at $1.77/kg.

### Strain engineering

The *GAL80* gene was deleted from strain Sp_Bc using the Cre/loxP recombination system, as detailed in previous work (Gueldener et al. 2002; Watcharawipas et al. 2021). A DNA donor sequence featuring loxP-URA3-loxP flanked by 50-bp homologous regions adjacent to the *GAL80* locus was first generated. This donor sequence was amplified using primers delGal80-F (5’AATCTCGATAGTTGGTTTCCCGTTCTTTCCACTCCCGTCAT GCATAGGCCACTAGTGGAT 3’) and delGal80-R (5’ GTTCGCTGCACTGGGGGCCA AGCACAGGGCAAGATGCTTTTACAGCTGAAGCTTCGTACG 3’), with the pUG72 plasmid serving as the template. Following amplification, the DNA was purified by gel extraction using the Qiagen Gel Extraction kit (Qiagen). Sp_Bc competent cells were then transformed with the purified donor DNA, and transformants were selected on SC medium deficient in uracil.

Confirmation of successful transformation involved colony PCR using primers deltaGal80-seq-5end-F (5’ AAGAAAATCACACGAGCG 3’) and Ura3-int-R (5’ AATTG GTCTTCTTTTCATCC 3’) for 5’ end verification, alongside Ura3-F (5’ ATGTCCACAA AATCATATACC 3’) and deltaGal80-seq-5end-R (5’ TCAGAACAAGA AATGATATGG 3’) for 3’ end verification). Subsequently, the *URA3* marker was removed from Sp_Bc ∆*gal80* cells by transformation with the pSH-Hyg plasmid, which encodes the Cre recombinase enzyme to facilitate marker excision. The pSH-Hyg plasmid was subsequently eliminated from the clones by repeated culturing in YPD medium devoid of hygromycin B. Only those clones that exhibited growth on SC medium containing 1 mg/mL 5-fluoroorotic acid, indicative of successful marker removal, and failed to grow on YPD medium with 200 µg/mL hygromycin B, confirming plasmid loss, were considered successful transformants.

### Small-scale β-carotene production

For shake-flask fermentation of strains Sp_Bc and Sp_Bc ∆*gal80*, the strains were pre-cultured in 25-mL aliquots in YPD medium (1% yeast extract, 2% peptone, and 2% glucose) overnight and used to inoculate 250 mL fresh YP medium containing the selected carbon source in 1000-mL shake flasks to achieve an initial OD_600_ of 0.1. The two carbon sources were 2% sucrose or 2% mixed carbon source (sucrose and galactose at a ratio of 1:2). The cultures were grown at 30 °C and 220 rpm in an orbital shaking incubator. Samples were taken at 0, 6, 12, 24, 48, 72, 96 and 120 h to determine OD_600_, dry cell weight (DCW), extracellular metabolites, and production of β-carotene.

### HPLC analysis of carotenoids and other cellular metabolites

Carotenoid extraction from engineered yeast strains was performed as previously reported (Watcharawipas et al. 2021). Carotenoid quantification was performed using the HPLC system (Vanquish) equipped with the Hypersil GOLD™ C18 column (4.6 mm x 150 mm) as previously reported (Watcharawipas et al. 2021). The liquid chromatography (LC) program utilized a mobile phase composed of acetonitrile, methanol, and isopropanol in a volumetric ratio of 5:3:2 at a flow rate of 1.2 mL/min. The column temperature was maintained at 20 °C. β-carotene was identified using a diode array detector (DAD) set to a visible light detection mode at 450 nm wavelength. Reference standard for β-carotene (catalog number PHR1239, Sigma-Aldrich) was prepared according to the manufacturer’s instructions. Representative chromatograms and UV–visible spectra of β-carotene peaks from the authentic standard and selected fermentation samples are provided in Figure S2 in the Supplementary Materials. Additionally, the quantification of compounds such as sucrose, fructose, glucose, glycerol, and ethanol was conducted using the HPLC system (Vanquish) equipped with a Shodex SH1011 column (8.0 mm x 300 mm). For this procedure, a 3 mM perchloric acid solution served as the mobile phase, maintained at a flow rate of 0.4 mL/min. The column temperature was maintained at 30 °C. The aforementioned compounds were identified using a refractive index detection (RID) system. All statistical analysis was performed using a two-tail, unpaired, heteroscedastic Student’s *t*-test in Microsoft Excel for Mac (Version 16.97.2).

### 5-L scale bioprocess development with refined sugars

Fed-batch fermentation was optimized in a 5-L stirred-tank bioreactor (Biostat B; Sartorius, Göttingen, Germany), following an adapted protocol from Watcharawipas et al. (Watcharawipas et al. 2021). The process initiated with 2 L of semi-defined medium, comprising 5 g/L ammonium sulfate, 3 g/L potassium dihydrogen phosphate, 0.5 g/L magnesium sulfate heptahydrate, 5 g/L yeast extract, 10 g/L peptone, and 1 mL/L trace metal solution (pH adjusted to 4.0). The trace metal solution contained 15.0 g/L EDTA (sodium salt), 4.5 g/L zinc sulfate heptahydrate, 1 g/L manganese(II) chloride tetrahydrate, 0.3 g/L cobalt(II) chloride hexahydrate, 0.3 g/L copper(II) sulfate pentahydrate, 0.4 g/L sodium molybdate dihydrate, 3.0 g/L calcium chloride dihydrate, 3.0 g/L iron(II) sulfate heptahydrate, 1.0 g/L boric acid, and 0.10 g/L potassium iodide. Sucrose (Mitr Phol: $0.70/kg) and galactose (Difco: $252.45/kg) were used as carbon sources. The batch phase included 20 g/L sucrose, while the fed-batch phase utilized sugar solutions with 500 g/L total sugars—either as a mixed solution (250 g/L sucrose and 250 g/L galactose) or solely sucrose. Seed cultures, grown to an OD_600_ of 5.5, were inoculated into the bioreactor to achieve an initial OD_600_ of 0.6. pH adjustment during fermentation was done using 2 N potassium hydroxide to maintain pH at 5.0. Fed-batch stages were initiated at the 10th hour with periodic sugar and yeast extract additions at 5-hour intervals over 20 cycles, reaching final concentrations of 120 g/L sugar and an additional 15 g/L yeast extract, totaling 30 g/L nitrogen source when combined with the initial 5 g/L yeast extract and 10 g/L peptone used in the batch phase. Sugar concentrations were controlled during the fed-batch phase to achieve optimal levels (keeping sucrose below 2.5 g/L and galactose below 2.5 g/L). Aeration was set at 1.0 VVM, with agitation at 500 rpm, and a constant temperature of 30 °C was maintained for optimal fermentation conditions.

### 2.6 Bioprocess development using M-molasses as carbon source

The bioprocess for β-carotene production using M-molasses as the carbon source was conducted in a 5-L stirred-tank bioreactor (Sartorius, Göttingen, Germany), paralleling the methods in Sect. 2.5 with the primary alteration being the replacement of refined sugars. M-molasses (Mitr Phol: $0.56/kg) was singularly used, providing 120 g/L total sugars in the fermentation medium. The operational parameters, including nutritional supplementation and environmental conditions, were consistent with those established in the refined sugar process.

### Bioprocess development using fish meal as nitrogen source

Adapting the fed-batch fermentation from Sect. 2.5, fish meal (High-Quality Fish Meal: $1.77/kg) was evaluated as an alternative nitrogen source in the 5-L Biostat B bioreactor (Sartorius). The substitution aimed to determine the efficacy of fish meal compared to traditional nitrogen sources, specifically yeast extract (Himedia: $62.27/kg) and peptone (Himedia: $71.81/kg). Trials were conducted with varying fish meal ratios, replacing the combined standard nitrogen sources at ratios of 100:0, 90:10, 80:20, and 70:30 (fish meal to yeast extract plus peptone). The ratio of yeast extract to peptone was consistent with the specifications detailed in Sect. 2.5 for both batch and fed-batch phases.

## Results and discussion

### GAL80 deletion and its impact on β-Carotene production

The parental strain used in this study, Sp_Bc, was previously engineered in our laboratory to produce β-carotene through multi-copy chromosomal integration of mevalonate pathway genes and carotenogenic genes (*SpCrtE*, *SpCrtYB*, and *SpCrtI* from *Sporidiobolus pararoseus*) under the control of *GAL1*–*GAL10* promoters (Watcharawipas et al. 2021). This configuration increases precursor supply and channels metabolic flux toward β-carotene biosynthesis but requires galactose for pathway induction. To enhance the cost-effectiveness of β-carotene production in *S. cerevisiae*, the deletion of the *GAL80* gene represented an important modification. The rationale for this modification lies in the high cost of galactose, which, despite being an effective inducer of the *GAL* promoter, presents a major barrier to economic scalability (Kim et al. 2022).

*GAL80* encodes a transcriptional repressor that regulates the cell’s transcription response to galactose (Timson et al. 2002). The regulatory mechanism in *S. cerevisiae* involves Gal80p’s interaction with the *GAL* promoters; in the presence of galactose, Gal80p is sequestered away from Gal4p, lifting the repression and activating the promoter (Weinhandl et al. 2014). Conversely, glucose—and by extension, sucrose—can repress this system by inhibiting *GAL* promoters through Mig1p and Mig2p (Ostergaard et al. 2000; Lim et al. 2011). The deletion of *GAL80* uncouples β-carotene production from the need for galactose, thereby enabling the use of sucrose as a cost-effective alternative.

**Fig. 1:**
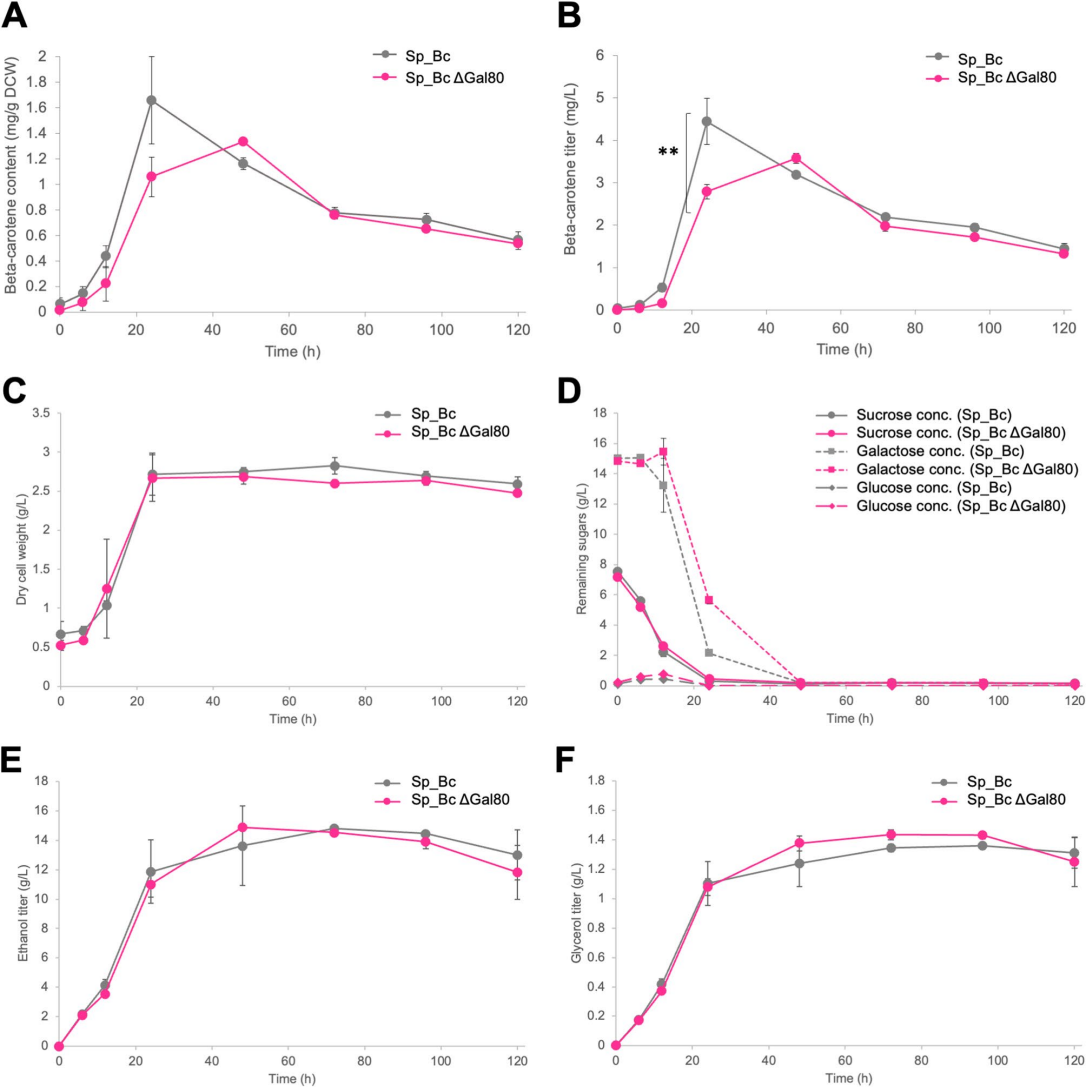
Comparative shake-flask fermentation profiles of β-carotene production in strains Sp_Bc and Sp_Bc ∆*gal80* in yeast medium containing a mixed carbon source (sucrose and galactose at 1:2 ratio, 2% total sugar concentration). Experiments were conducted in triplicate and values are presented as mean ± standard deviation. (**A**) β-carotene content (mg/g DCW); (**B**) β-carotene titer (mg/L); (**C**) Biomass accumulation as dry cell weight (g/L); (**D**) Remaining sugar levels (sucrose, galactose, and glucose); (**E**) Ethanol titer (g/L); and (**F**) Glycerol titer (g/L). Statistical analysis was performed using a two-tail, unpaired, heteroscedastic Student’s *t*-test. ** *P* < 0.05

Deletion of *GAL80* significantly altered β-carotene production in Sp_Bc ∆*gal80* versus wild-type Sp_Bc (Fig. 1; see Supplementary Materials). With a mixed carbon source, wild-type Sp_Bc began β-carotene production immediately, while Sp_Bc ∆*gal80* showed a 6-hour delay, likely due to pre-culture conditions inducing membrane stress (Fig. 1B) (Verwaal et al. 2010). In the 24 h after initiation, the modified strain Sp_Bc ∆*gal80* gradually increased its β-carotene content to 1.06 ± 0.16 mg/g DCW at 24 h; although this was notably lower than wild-type Sp_Bc’s peak of 1.66 ± 0.34 mg/g DCW. However, by the 48-hour mark, the modified Sp_Bc ∆*gal80*’s content surpassed that of the wild-type Sp_Bc, reaching 1.33 ± 0.03 mg/g DCW and 1.16 ± 0.05 mg/g DCW, respectively.

Both strains experienced β-carotene content decline, consistent with photodegradation (Scita 1992; Liu and Kong 2020). While supplementing with antioxidants such as butylated hydroxytoluene (BHT) could mitigate this degradation (Park et al. 2022), it is important to note that in an industrial setting, microbial cultures are typically conducted in stainless steel vessels. These opaque containers inherently shield cultures from light exposure, thus naturally minimizing the risk of photo-oxidation without the need for dark fermentation conditions, which simplifies operational processes and reduces potential infrastructural complexities.

Ethanol and glycerol levels, by-products of yeast metabolism (van Aalst et al. 2022), remained similar for both strains, indicating that *GAL80* deletion did not affect metabolic flux toward these compounds (Fig. 1E and F). Ethanol decreased after 48–72 h, and glycerol production stabilized, demonstrating a resilient metabolic network despite *GAL80* deletion.

**Fig. 2:**
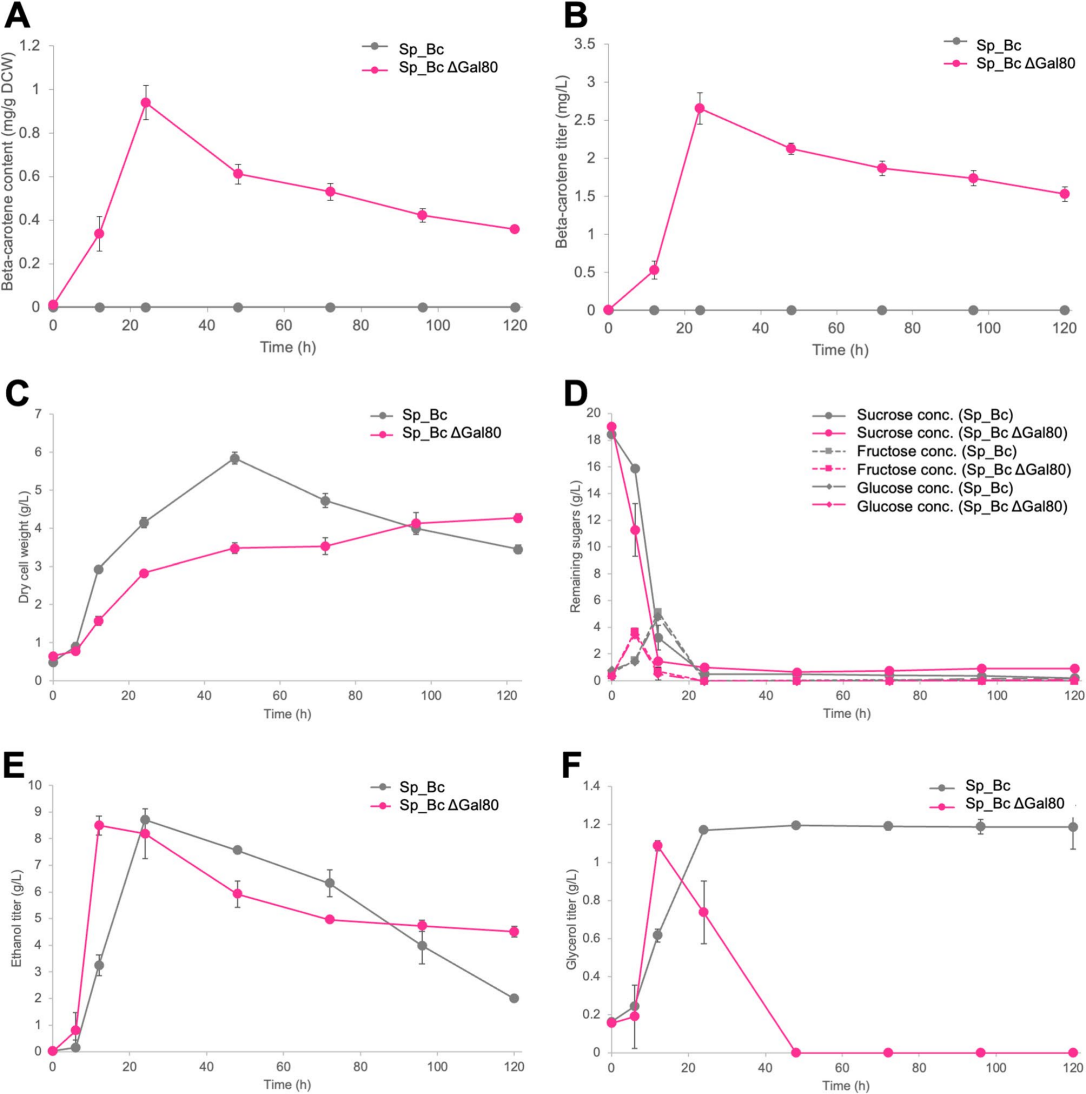
Comparative shake-flask fermentation profiles of β-carotene production in strains Sp_Bc and Sp_Bc ∆*gal80* in yeast medium containing 2% sucrose as the sole carbon source. Experiments were conducted in triplicate and values are presented as mean ± standard deviation. (**A**) β-carotene content (mg/g DCW); (**B**) β-carotene titer (mg/L); (**C**) Biomass accumulation as dry cell weight (g/L); (**D**) Remaining sugar levels (sucrose, fructose, and glucose); (**E**) Ethanol titer (g/L); and (**F**) Glycerol titer (g/L)

When sucrose was the sole carbon source, Sp_Bc did not produce detectable β-carotene, as *GAL1/10/7* promoter-controlled genes require galactose for induction (Fig. 2). Sp_Bc ∆*gal80*, engineered to bypass galactose-dependent regulation, produced measurable amounts of β-carotene, with a peak content of 0.94 ± 0.08 mg/g DCW and titer of 2.66 ± 0.21 mg/L at 24 h (Fig. 2A and B). However, there was a subsequent decrease, mirroring mixed carbon source findings.

The Sp_Bc ∆*gal80* strain showed reduced biomass compared to Sp_Bc (Fig. 2C). This observation suggests that β-carotene biosynthesis may lead to an energy drain, acetyl-CoA redirection away from cellular growth, and potential redox imbalances. These metabolic costs, in turn, could contribute to the reduced growth and biomass observed in the β-carotene-producing Sp_Bc ∆*gal80* strain. Moreover, the overproduction of β-carotene, a lipophilic compound, can integrate into cell membranes, potentially compromising membrane integrity and leading to cell stress or death, thereby impacting biomass accumulation (Gao et al. 2017; Jing et al. 2022).

In sucrose fermentation, Sp_Bc ∆*gal80* demonstrated an initial increase in ethanol production, with a slightly elevated level in later stages, possibly indicating sustained fermentation due to metabolic rerouting. Glycerol production in Sp_Bc ∆*gal80* peaked early and ceased by 48 h. Glycerol is typically produced by yeast as a response to osmotic stress and as a means to maintain redox balance by regenerating NAD^+^ from NADH during anaerobic conditions (Lambrechts and Pretorius 2000; Petelenz-Kurdziel et al. 2013; Duncan et al. 2023). Its early peak in the Sp_Bc ∆*gal80* strain may indicate an initial osmotic response due to the high sucrose concentration. The subsequent cessation of glycerol production could suggest an adaptive response or a shift in metabolic focus towards β-carotene synthesis, which may alter the intracellular redox potential and reduce the necessity for NADH reoxidation, thus impacting glycerol accumulation. This shift could also reflect the metabolic adjustments the engineered strain undergoes to accommodate β-carotene production, prioritizing NADPH-dependent biosynthetic pathways over glycerol formation for redox balancing. Collectively, these experiments show that Sp_Bc ∆*gal80* efficiently synthesizes β-carotene from sucrose, leveraging its ability to alleviate glucose repression on *GAL* promoters.

### Bioprocess development using sucrose as a carbon source

**Fig. 3:**
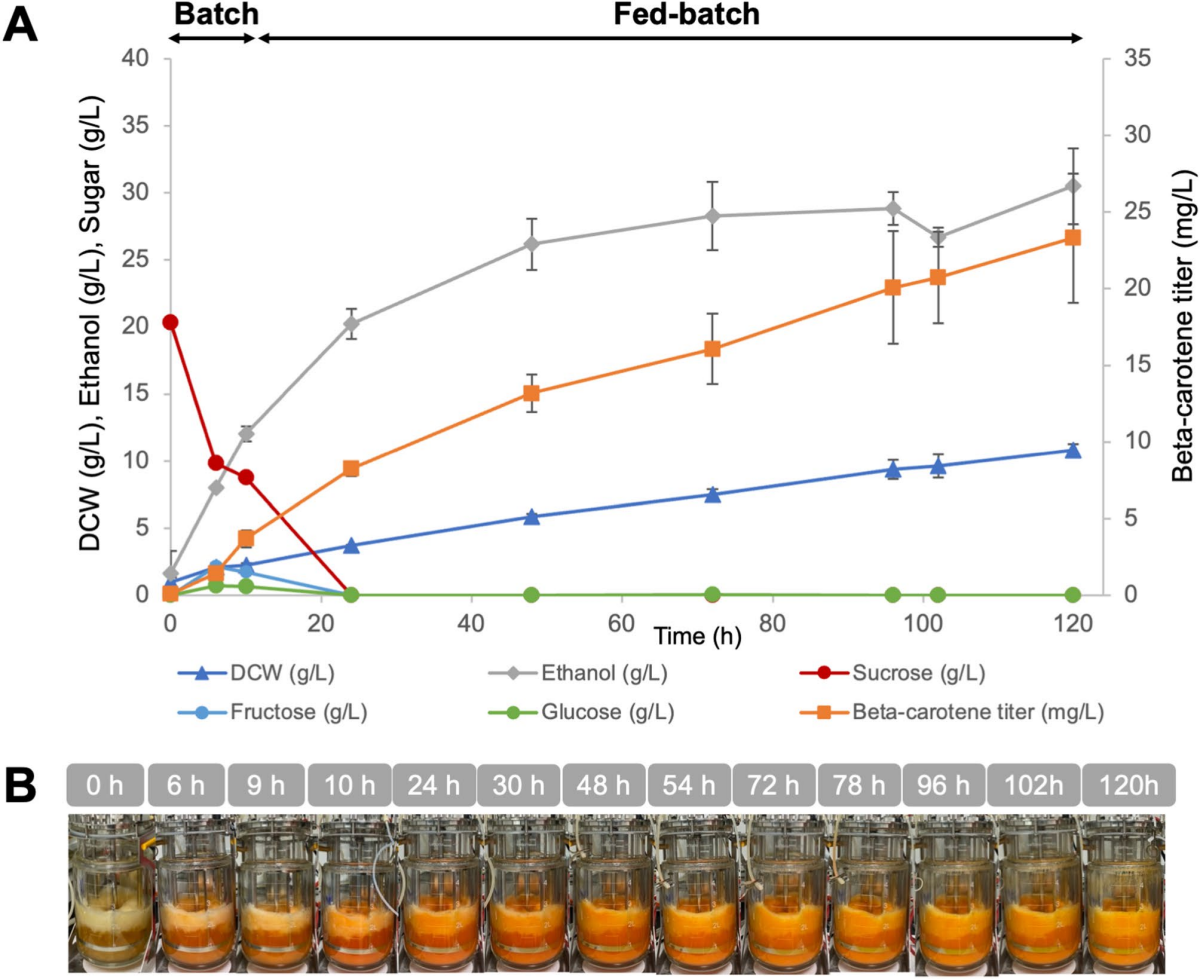
Fed-batch fermentation of Strain Sp_Bc ∆*gal80* in a 5-L fermenter using sucrose as the sole carbon source. Fermentation profile including β-carotene titer, biomass as dry cell weight (DCW), ethanol production, and residual sugars over time, with averages and standard deviations from duplicate runs. (**B**) Time-lapse visualization of Sp_Bc ∆*gal80* culture in a bioreactor with sucrose

The performance of strain Sp_Bc ∆*gal80* observed in shake-flask fermentation justified the upscaling of β-carotene production to a 5-L stirred-tank bioreactor, using sucrose as the sole carbon source (Fig. 3). The strategic shift from previous methods that combined sucrose and galactose (Watcharawipas et al. 2021) aimed to streamline the carbon source, reducing overall production costs and simplifying the fermentation process. The batch phase commenced with 20 g/L sucrose in a semi-defined medium. To avoid the potential inhibitory effects of high sucrose concentrations, the fed-batch stage was carefully managed with periodic sucrose additions. These were scheduled at 5-hour intervals across 20 cycles, ensuring optimal sugar levels for sustained β-carotene biosynthesis without compromising cell health.

Throughout fermentation, the Sp_Bc ∆*gal80* strain effectively converted sucrose into β-carotene, achieving a maximum titer of 23.30 ± 4.22 mg/L at 120 h (Fig. 3). The identity of β-carotene in the fed-batch samples was confirmed by comparison of the retention time and UV-visible spectrum with an authentic standard (see Figure S2 in Supplementary Materials). For context, recent high-performance *S. cerevisiae* systems optimized for β-carotene production have achieved titers up to 2.09 g/L in a 5-L fed-batch bioreactor, following extensive multi-layer metabolic engineering, pathway copy-number optimization, lipid droplet remodeling, and even atmospheric and room temperature plasma (ARTP) mutagenesis to generate hyper-producing strains (Lin et al. 2025). Such strategies substantially reconfigure central metabolism, precursor supply, and carotenoid storage capacity to maximize flux toward the product.

In contrast, the strain in this study was developed through a more targeted approach, combining GAL-promoter-driven expression of heterologous carotenoid biosynthetic genes with deletion of the *GAL80* repressor, without broader genome-wide optimization or adaptive mutagenesis. Given this difference in engineering scope, it is not surprising that our absolute titer is lower than those reported from heavily optimized laboratory strains. However, the present work addresses a different but equally important challenge: demonstrating that industrially relevant and low-cost carbon sources such as sucrose and M-molasses can be directly converted to β-carotene in an engineered *S. cerevisiae* without the need for galactose induction or expensive refined substrates.

The β-carotene content achieved by the Sp_Bc ∆*gal80* strain reached a peak of 2.29 ± 0.16 mg/g DCW at 48 h and maintained a content of 2.15 ± 0.46 mg/g DCW at 120 h, exceeding that of traditional plant-based sources such as carrots (∼ 0.52 mg/g DCW) (Dias et al. 2018). Ethanol production peaked at 28.3 ± 2.6 g/L and plateaued after 72 h. Residual sugar analysis revealed complete sucrose utilization, with no fructose or glucose detected after 24 h, indicating an efficient metabolic flux towards β-carotene synthesis. This is in stark contrast to the shake-flask experiments, where residual sugars persisted longer, and β-carotene production appeared to exert a metabolic cost on the cells. The bioreactor environment seemingly provided a more supportive backdrop for the Sp_Bc ∆*gal80* strain to overcome the challenges of β-carotene toxicity. This could be due to enhanced cellular mechanisms for managing oxidative stress or a more efficient partitioning of metabolic resources between growth and product formation under controlled fermentation conditions.

This foundation leaves ample scope for performance enhancement. Future work could integrate targeted precursor pathway engineering, lipid droplet capacity expansion, dynamic pathway regulation, and adaptive laboratory evolution to increase yield while retaining compatibility with low-cost feedstocks. The combination of substrate cost savings and strain performance improvements would further strengthen the economic viability of β-carotene production at industrial scale.

### Molasses as an alternative carbon source: β-carotene production and comparison with sucrose

The exploration of cost-effective carbon sources for β-carotene production in engineered *S. cerevisiae* identified molasses, a dense, sugar-rich by-product of sugar refining, as a viable option. Recognized for its cost-efficiency and sustainability, molasses has been employed in bio-production processes including biofuels and bioplastics, substantiating its versatility (Tripathi et al. 2012; Anjali et al. 2014; Gonçalves et al. 2016). Particularly for β-carotene production, its abundant sugar content offers a promising alternative to sucrose, potentially enhancing the economic feasibility of industrial applications.

**Fig. 4:**
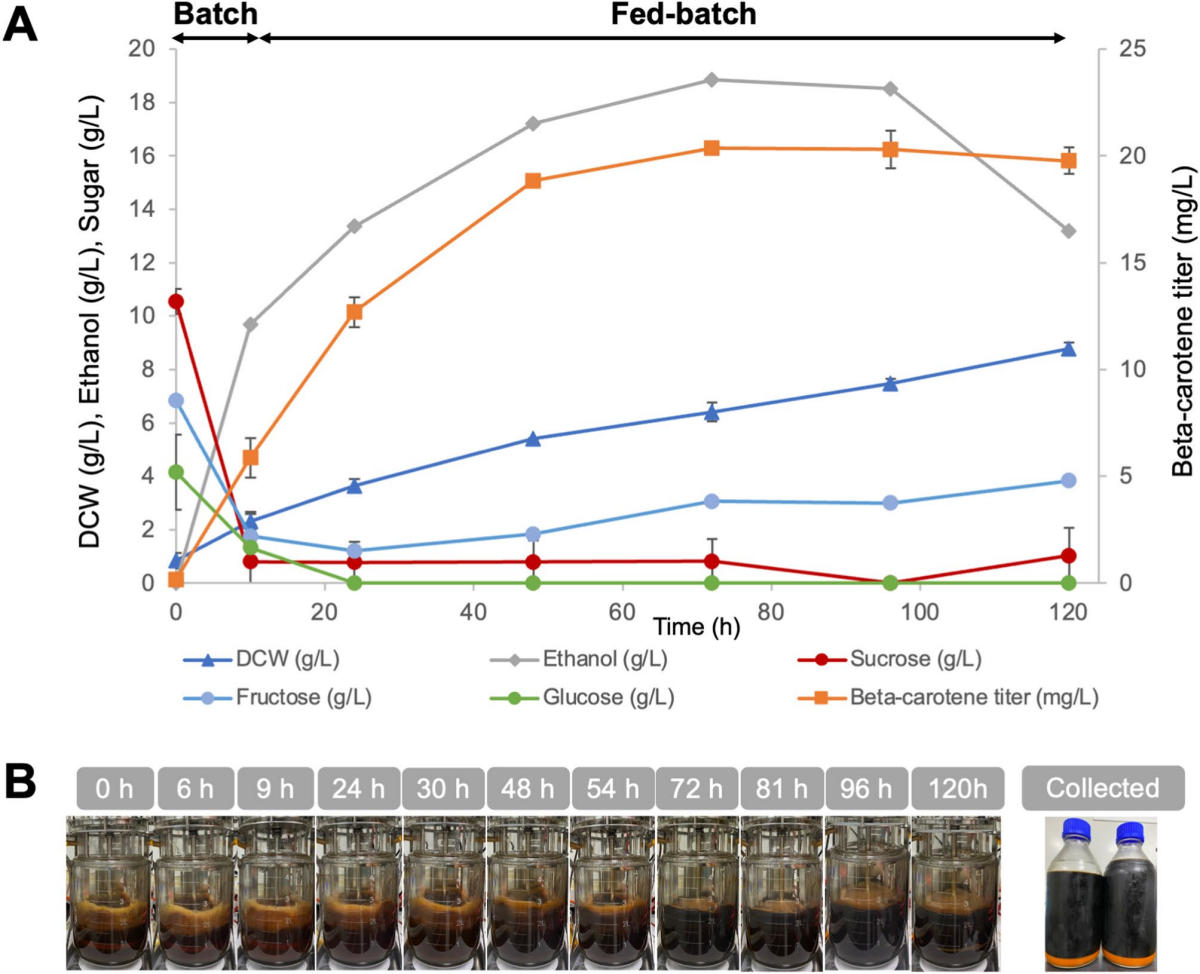
Fed-batch fermentation of Strain Sp_Bc ∆*gal80* in a 5-L fermenter using M-molasses as the sole carbon source. (**A**) Fermentation profile including β-carotene titer, biomass as dry cell weight (DCW), ethanol production, and residual sugars over time, with averages and standard deviations from duplicate runs. (**B**) Time-lapse visualization of Sp_Bc ∆*gal80* culture in a bioreactor with M-molasses

Experiments in a 5-L stirred-tank fermenter employed M-molasses as the sole carbon source, added at a concentration to provide 120 g/L of total fermentable sugars, matching the sugar level used in sucrose-based fermentation. Under these conditions, the engineered strain achieved a β-carotene titer of 20.36 ± 0.08 mg/L at 72 h (Fig. 4). A slight decline was observed by the 120-hour mark, which may reflect product degradation as reported previously (Scita 1992; Liu and Kong 2020). The peak β-carotene content was 3.50 ± 0.43 mg/g DCW at 24 h, comparable to the values obtained from sucrose-based media fermentation, demonstrating the feasibility of M-molasses as a carbon source.

However, compared to sucrose-based media, M-molasses did result in a lower β-carotene titer (maximum titers of 20.36 ± 0.08 mg/L for M-molasses vs. 23.30 ± 4.22 mg/L for sucrose; Figs. 3 and 4). This disparity may stem from the complex composition of M-molasses, which contains a variety of sugars and impurities that can affect yeast metabolism and stress response, potentially leading to decreased β-carotene synthesis (Lee et al. 2023). To address this, strategies such as pretreatment of molasses to reduce impurities and further genetic engineering to enhance yeast’s tolerance to pretreatment inhibitors can be explored (Lopez-Arenas et al. 2022).

Importantly, the ‘dirtiness’ of M-molasses does not translate to complications in product purity (Fig. 4B). β-carotene is predominantly sequestered within the yeast cells, negating the need for extensive purification processes to separate the product from the culture medium (Bu et al. 2020). This intracellular storage is advantageous, as it simplifies downstream processing, further reducing production costs and potentially increasing the overall process sustainability.

This study represents, to the authors’ knowledge, the first successful demonstration of engineered *S. cerevisiae* utilizing M-molasses as the primary carbon source for β-carotene synthesis. Previous studies employing molasses for carotenoid production typically involved naturally carotenogenic yeast species, such as *Rhodotorula glutinis* and *Rhodotorula mucilaginosa*, achieving carotenoid titers ranging from approximately 3.7 mg/L up to 125 mg/L of total carotenoids (Bhosale and Gadre 2001; Aksu and Eren 2007; Rodrigues et al. 2019). However, direct comparisons to these studies are challenging, as they report total carotenoids rather than β-carotene specifically, and employ species naturally capable of carotenoid biosynthesis.

Despite lower titers relative to some reported microbial systems, the current approach highlights significant economic and sustainability advantages associated with using inexpensive, widely available agricultural by-products such as molasses. These benefits may outweigh higher titers achieved using refined substrates when considering industrial scalability and overall production costs. With further process optimization, including pretreatment of molasses and targeted genetic improvements to enhance substrate utilization efficiency, the engineered *S. cerevisiae* strain developed in this study has substantial potential for cost-effective industrial-scale β-carotene production.

### Fish meal as an alternative nitrogen source: effects of varying fish meal ratios on β-carotene production

The preceding section outlined how M-molasses serves as an efficient alternative carbon source for β-carotene production in engineered *S. cerevisiae*. Following this, the focus shifted to the nitrogen source to further enhance the sustainability and cost-effectiveness of the production medium. Fish meal, obtained from fish industry by-products such as bone, viscera, and processing residues, offers a rich array of nutrients essential for yeast growth. These by-products are generated in substantial quantities in major aquaculture-producing countries, including Thailand and China, and their valorization aligns with circular economy principles by reducing waste and creating value-added products.

Nutritional analyses of fish waste-derived hydrolysates have shown that they contain bioavailable peptides, amino acids, vitamins, and minerals that can enhance microbial biomass formation and modulate metabolic pathways (Zhang et al. 2024). In *Bifidobacterium animalis* subsp. *lactis* BB-12, for example, Zhang et al. (2024) demonstrated that fish waste hydrolysates improved both growth and metabolite production, suggesting similar potential benefits for carotenoid biosynthesis in yeast. The success of using fish meal as a nitrogen source is further supported by a recent work of Xiong et al. (2024), who demonstrated the potential of fish bone meal enzymatic hydrolysate in the production of ergothioneine, a valuable antioxidant, with *Rhodotorula mucilaginosa* (Xiong et al. 2024). This study not only achieved a substantial ergothioneine yield of 216.3 mg/L through fed-batch fermentation but also significantly reduced the feedstock costs by 330.9% compared to traditional YPD media. Together, these studies highlight fish meal as an attractive, low-cost nitrogen source with demonstrated potential to support high-value biochemical production.

**Fig. 5:**
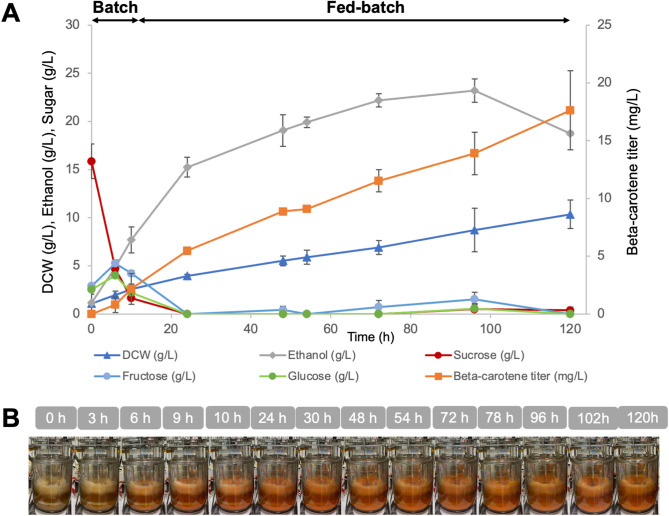
Fed-batch fermentation of Strain Sp_Bc ∆*gal80* in a 5-L fermenter using fish meal as an alternative nitrogen source. Fermentation profile including β-carotene titer, biomass as dry cell weight (DCW), ethanol production, and residual sugars over time, with averages and standard deviations from duplicate runs. Data points represent the mean values obtained from duplicate fermentations, with error bars indicating the standard deviation. (**B**) Visual progression of the bioreactor culture over time

The impact of various fish meal to yeast extract and peptone (FE: YE + P) ratios on β-carotene production was assessed. The ratios were: (i) 100% Fish Extract (FE), (ii) 90:10 (FE: YE + P), (iii) 80:20 (FE: YE + P) and (iv) 70:30 (FE: YE + P). The 5-L fermenter results demonstrated that complete replacement with fish meal (100:0) led to a substantial decrease in β-carotene production (maximum titer and content of 11.62 ± 1.00 mg/L and 1.75 ± 0.15 mg/g DCW, respectively), suggesting that some components essential for optimal yeast performance might be missing in fish meal (see Supplementary Materials). At a 90:10 ratio, β-carotene production was still lower than desired (maximum titer and content of 16.02 ± 2.10 mg/L and 1.95 ± 0.02 mg/g DCW, respectively). An 80:20 fish meal to yeast extract and peptone ratio showed a β-carotene titer of 17.63 ± 3.42 mg/L and a content of 1.70 ± 0.09 mg/g DCW (Fig. 5). The 70:30 ratio resulted in further improvements (maximum β-carotene titer and content of 21.46 ± 2.10 mg/L and 2.21 ± 0.05 mg/g DCW, respectively). The 80:20 fish meal to yeast extract and peptone ratio was selected for further optimization based on its performance, indicating that a significant proportion of traditional nitrogen sources could be replaced without compromising β-carotene production.

While these results are promising, they fall short when compared to a control fermentation using yeast extract and peptone, which achieved a β-carotene titer and content of 23.30 ± 4.22 mg/L and 2.29 ± 0.16 mg/g DCW, respectively (Fig. 3). Several factors could contribute to the lower yield observed with fish meal. The quality and composition of nitrogen in fish meal may not be as readily assimilated by yeast cells as the nitrogen from yeast extract and peptone. Additionally, fish meal might contain other components that could inhibit yeast metabolism or β-carotene biosynthesis. It’s also possible that the metabolic burden of processing the nitrogen from fish meal could divert energy from β-carotene synthesis to growth and maintenance functions within the cells. Future investigations could focus on refining the preparation and processing of fish meal to further improve its utility as a nitrogen source (Xiong et al. 2024). Examining fish meal treatment to boost the availability of assimilable nitrogen, and supplementing it with essential micronutrients found in yeast extract and peptone, could improve outcomes.

### Combination of M-molasses as an alternative carbon source and fish meal as an alternative nitrogen source

**Fig. 6:**
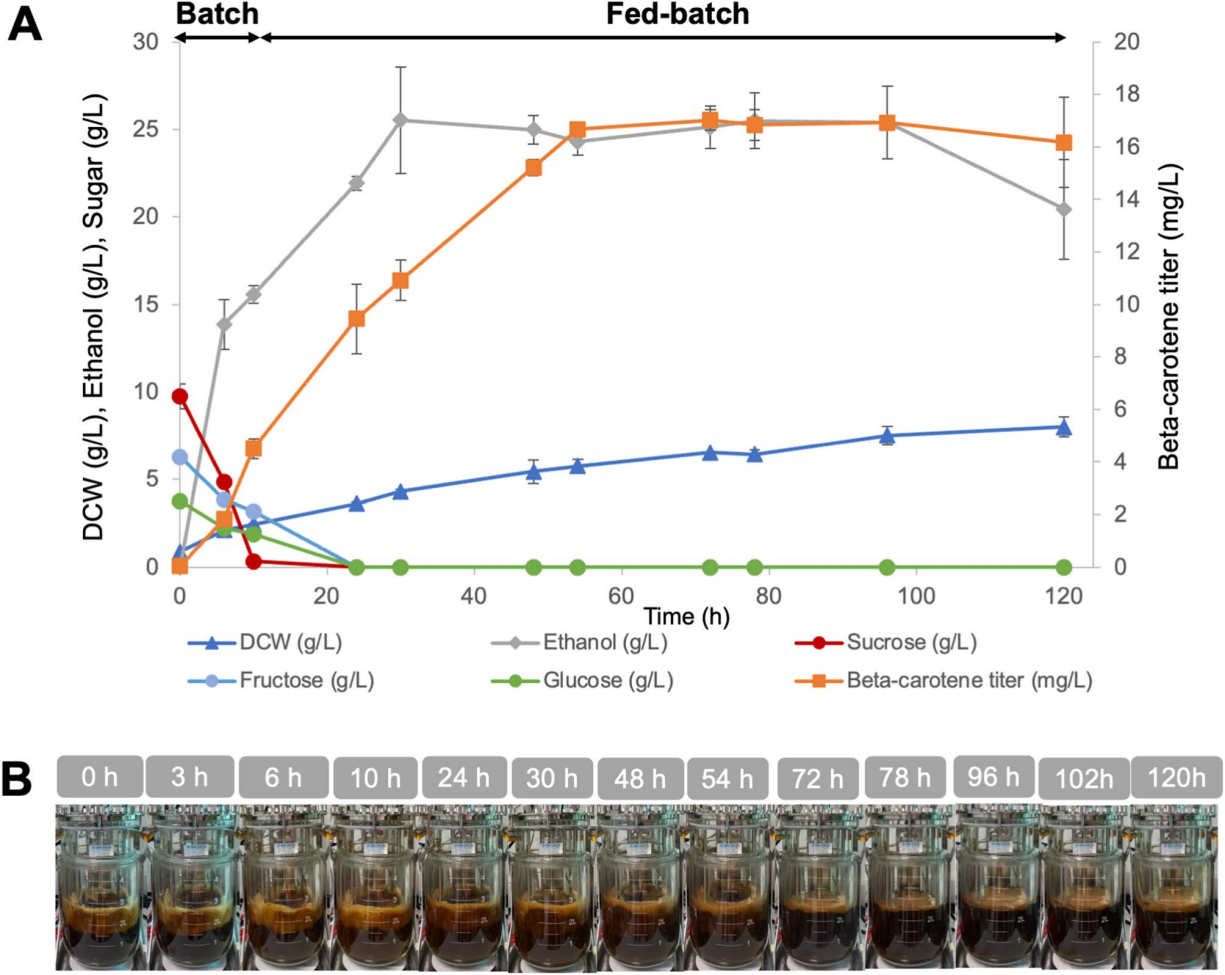
Fed-batch fermentation of Strain Sp_Bc ∆*gal80* in a 5-L fermenter using fish meal and M-molasses as alternative nitrogen and carbon sources, respectively. Fermentation profile including β-carotene titer, biomass as dry cell weight (DCW), ethanol production, and residual sugars over time, with averages and standard deviations from duplicate runs. Data points represent the mean values obtained from duplicate fermentations, with error bars indicating the standard deviation. (**B**) Visual progression of the bioreactor culture over time

The study built upon previous findings, using M-molasses and fish meal as simultaneous substitutes for traditional carbon and nitrogen sources in *S. cerevisiae* fermentation, which could enhance the cost-efficiency and sustainability of β-carotene production. The current experiment replaced 80% of the total nitrogen source with fish meal, achieving a concentration of 24 g/L from the total 30 g/L provided. M-molasses was used as the sole carbon source, added at a concentration that provided 120 g/L total fermentable sugars in the medium. The combined effect of these substitutions was assessed in a 5-L fermenter across a 120-hour fermentation cycle. The fermentation culminated with a dried cell weight of 8.0 ± 0.6 g/L, and the β-carotene titer peaked at 17.02 ± 0.40 mg/L after 72 h, marginally reducing to 16.18 ± 1.72 mg/L by the conclusion of the fermentation (Fig. 6). Notably, the highest β-carotene content achieved was 2.90 ± 0.21 mg/g DCW at the 54-hour mark, demonstrating the potential of integrating alternative raw materials in the production medium.

These results are promising and may represent the first successful endeavor to produce β-carotene in *S. cerevisiae* using agricultural waste products for both carbon and nitrogen sources, according to the available literature. While the β-carotene titer and content did not reach the levels obtained with sucrose, they are a promising starting point for further improvement. Optimizing fermentation conditions, enriching M-molasses with targeted nutrients, and tailoring yeast strains for better assimilation of these complex substrates are clear pathways to enhanced β-carotene yields. There is considerable potential in further developing this process, with opportunities to reduce costs and improve the environmental footprint of industrial fermentation.

### Cost considerations for alternative carbon and nitrogen sources in β-Carotene production

The economic viability of alternative carbon and nitrogen sources for β-carotene production is critical for industrial scale-up. This study analyzed medium costs in USD, converted from Thai baht (exchange rate: 35.65 THB per 1 USD), to quantify potential cost reductions (Table 1 and Supplementary Excel File 1). Fermentation medium components, including carbon and nitrogen sources, are recognized as major contributors to operating costs in microbial fermentation processes, particularly in early-stage techno-economic analyses that focus on feedstock and nutrient expenses (Noroozi and Jarboe 2023; Etit et al. 2024). Employing fish extract entirely as a nitrogen source substantially lowered medium costs, reducing the medium cost from $2.21/L (sucrose with yeast extract and peptone) to $0.30/L (sucrose with fish extract alone). This dramatic cost reduction led to a corresponding decrease in the production cost of β-carotene from $94.84/g to $25.75/g, achieving approximately a 72.85% reduction.

**Table 1:** Comparative cost analysis of medium components for β-carotene production utilizing sp_bc ∆*gal80* strain in a 5-L fermenter, encompassing different carbon and nitrogen sources

Condition	Carbon source	Nitrogen source	Medium cost (THB/L)	Medium cost (USD/L)	β-carotene titer achieved (mg/L)	β-carotene production cost (THB/mg of β-carotene)	β-carotene production cost (THB/gram of β-carotene)	β-carotene production cost (USD/gram of β-carotene)
1	Sucrose	Yeast extract and peptone (100%)	78.77	2.21	23.30 ± 4.22	3.38	3380.89	94.84
2	Sucrose	Fish extract: yeast extract and peptone (70:30)	31.10	0.87	21.46 ± 2.10	1.45	1449.17	40.65
3	Sucrose	Fish extract: yeast extract and peptone (80:20)	24.29	0.68	17.63 ± 3.42	1.38	1377.73	38.65
4	Sucrose	Fish extract: yeast extract and peptone (90:10)	17.48	0.49	16.02 ± 2.10	1.09	1090.60	30.59
5	Sucrose	Fish extract (100%)	10.66	0.30	11.62 ± 1.00	0.92	917.87	25.75
6	Sucrose mix with galactose	Yeast extract and peptone (100%)	527.52	14.80	26.84 ± 2.20	19.66	19657.35	551.40
7	M-molasses	Yeast extract and peptone (100%)	81.33	2.28	20.36 ± 0.08	3.99	3994.73	112.05
8	M-molasses	Fish extract: yeast extract and peptone (80:20)	26.84	0.75	17.02 ± 0.40	1.58	1557.01	44.24

When M-molasses replaced sucrose as the carbon source (with yeast extract and peptone), medium costs remained relatively similar, approximately $2.28/L compared to $2.21/L for sucrose, leading to a slightly higher production cost of $112.05/g β-carotene. However, integrating M-molasses with a nitrogen source consisting of an 80:20 ratio of fish extract to yeast extract and peptone significantly decreased the medium cost to $0.75/L. This strategy notably reduced β-carotene production costs from $112.05/g to $44.24/g, marking an approximately 60.52% reduction compared to molasses with traditional nitrogen sources.

These findings illustrate that leveraging alternative, less costly substrates can drastically lower production expenses. For instance, substituting traditional nitrogen sources (yeast extract and peptone) with fish extract reduced β-carotene production costs across various sucrose-based conditions, with reductions of 59.24% (80:20 FE: YE), 67.76% (90:10 FE: YE), and 57.13% (70:30 FE: YE) compared to conventional yeast extract and peptone medium. Although downstream processing costs (e.g., extraction and purification) can also be significant—sometimes exceeding media costs in large-scale operations—medium costs often remain an influential factor in early-stage cost assessments for fermentation-based processes (Etit et al. 2024).

Although M-molasses underwent fewer refinement steps than sucrose, medium costs per liter remained slightly higher due to market factors including global sugar production dynamics, local taxation, and perceived value as a specialty substrate (Sowcharoensuk 2023). Nevertheless, the cost benefit becomes evident when combined with fish extract, underscoring the strategic potential for cost-sensitive industrial-scale β-carotene production.

It is important to note this cost analysis primarily addresses fermentation medium components and does not account for downstream processing expenses. However, since β-carotene is primarily accumulated intracellularly, medium impurity likely has minimal impact on downstream extraction efficiency, as cells can be efficiently separated via centrifugation, further supporting overall cost-effectiveness.

In summary, this study demonstrates that selecting alternative, economically favorable substrates—particularly fish extract for nitrogen and M-molasses for carbon—can significantly decrease β-carotene production costs. This approach aligns industrial biotechnology with sustainable economic practices, enhancing the market competitiveness and accessibility of microbial β-carotene production. Future research optimizing medium pretreatments and strain robustness can further enhance these economic advantages, providing a practical pathway toward affordable and sustainable industrial-scale β-carotene production.

Beyond demonstrating the feasibility of β-carotene production from low-cost industrial and agricultural feedstocks, the present work establishes a platform that can be incrementally improved through targeted metabolic engineering and process optimization. Potential enhancements include increasing precursor availability via fine-tuning of the mevalonate pathway, expanding intracellular lipid droplet capacity to improve carotenoid storage, and implementing dynamic pathway regulation to balance growth and production phases. Adaptive laboratory evolution and mutagenesis-based approaches could further improve substrate utilization efficiency and stress tolerance, particularly when using more complex feedstocks such as molasses or fish meal hydrolysates. Combining these strategies with the feedstock flexibility demonstrated here would enable the development of robust, high-yielding production strains suitable for industrial deployment, maximizing both economic viability and environmental sustainability.

## Conclusions

In this study, the engineered yeast strain Sp_Bc ∆*gal80* successfully produced β-carotene directly from sucrose, eliminating the costly requirement for galactose induction. Furthermore, this work demonstrated, for the first time, the feasibility of utilizing sustainable and inexpensive agricultural by-products—M-molasses and fish meal—as effective alternative carbon and nitrogen sources for β-carotene production. Incorporating these readily available substrates significantly reduced production medium costs by up to 73% compared to traditional yeast extract and peptone-based media. These findings underscore the considerable economic advantages and environmental sustainability associated with employing renewable agricultural resources in microbial β-carotene production. Ultimately, this approach presents a practical and environmentally responsible platform, highlighting strong potential for enhancing industrial production and creating accessible, sustainable supply chains for β-carotene and other valuable nutritional biochemicals.

## Supplementary Information

Below is the link to the electronic supplementary material.

Supplementary Material 1

Supplementary Material 2

## Data Availability

All data generated or analysed during this study are included in this published article and its supplementary information files. Further datasets from the current study can be obtained from the corresponding authors upon reasonable request.

## Abbreviations

DCW: Dry cell weight
YPD medium: Yeast extract-peptone-dextrose medium
SC medium: Synthetic complete medium
HPLC: High-performance liquid chromatography
LC: Liquid chromatography
DAD: Diode array detector
EDTA: Ethylenediaminetetraacetic acid
OD_600_: Optical density at 600 nm
VVM: Vessel volume/minute
BHT: Butylated hydroxytoluene
NAD^+^/NADH: Oxidized and reduced forms of nicotinamide adenine dinucleotide
NADPH: Nicotinamide adenine dinucleotide phosphate
FE: Fish extract/fish meal
YE: Yeast extract
