# Supplementary material for: Sustainable β-carotene production by engineered S. cerevisiae using sucrose and agricultural by-products
Source: Bioresour Bioprocess. 2025 Sep 13;12(1):96. doi: 10.1186/s40643-025-00936-y (PMC12433382; doi:10.1186/s40643-025-00936-y)
Supplement: Supplementary file 2 — Supplementary Material 2 [file 40643_2025_936_MOESM2_ESM.docx]

**Supporting Information for Sustainable β-Carotene Production by Engineered *S. cerevisiae* Using Sucrose and Agricultural By-Products**

**Table S1: Sugar composition of M-molasses**

**Figure S1:** Visual progression of shake-flask cultures in yeast medium. Panel A (top row: Sp_Bc, bottom row: Sp_Bc ∆*gal80*) with a mixed carbon source (sucrose and galactose at 1:2 ratio, 2% total sugar concentration) shows an intensifying orange hue, indicative of increasing beta-carotene concentration. Panel B (top row: Sp_Bc, bottom row: Sp_Bc ∆*gal80*) with 2% sucrose as the sole carbon source demonstrates the orange hue only in Sp_Bc ∆*gal80*, signaling beta-carotene production, while Sp_Bc lacks this hue, indicating its absence. All experiments were conducted in triplicate.

**Figure S2.** High-performance liquid chromatography (HPLC) chromatograms recorded at 450 nm for β-carotene analysis. **A**: β-carotene authentic standard. **B**: Sp_Bc *∆gal80* cultured in fed-batch mode with sucrose as carbon source and yeast extract (YE) + peptone as nitrogen source, sampled at 120 h. **C**: Sp_Bc *∆gal80* cultured in fed-batch mode with M-molasses as carbon source and YE + peptone as nitrogen source, sampled at 120 h. **D**: Sp_Bc *∆gal80* cultured in fed-batch mode with M-molasses as carbon source and fish extract:YE + peptone (80:20) as nitrogen source, sampled at 120 h. UV–visible spectra of the β-carotene peak for each sample are shown to the right of each chromatogram.

**
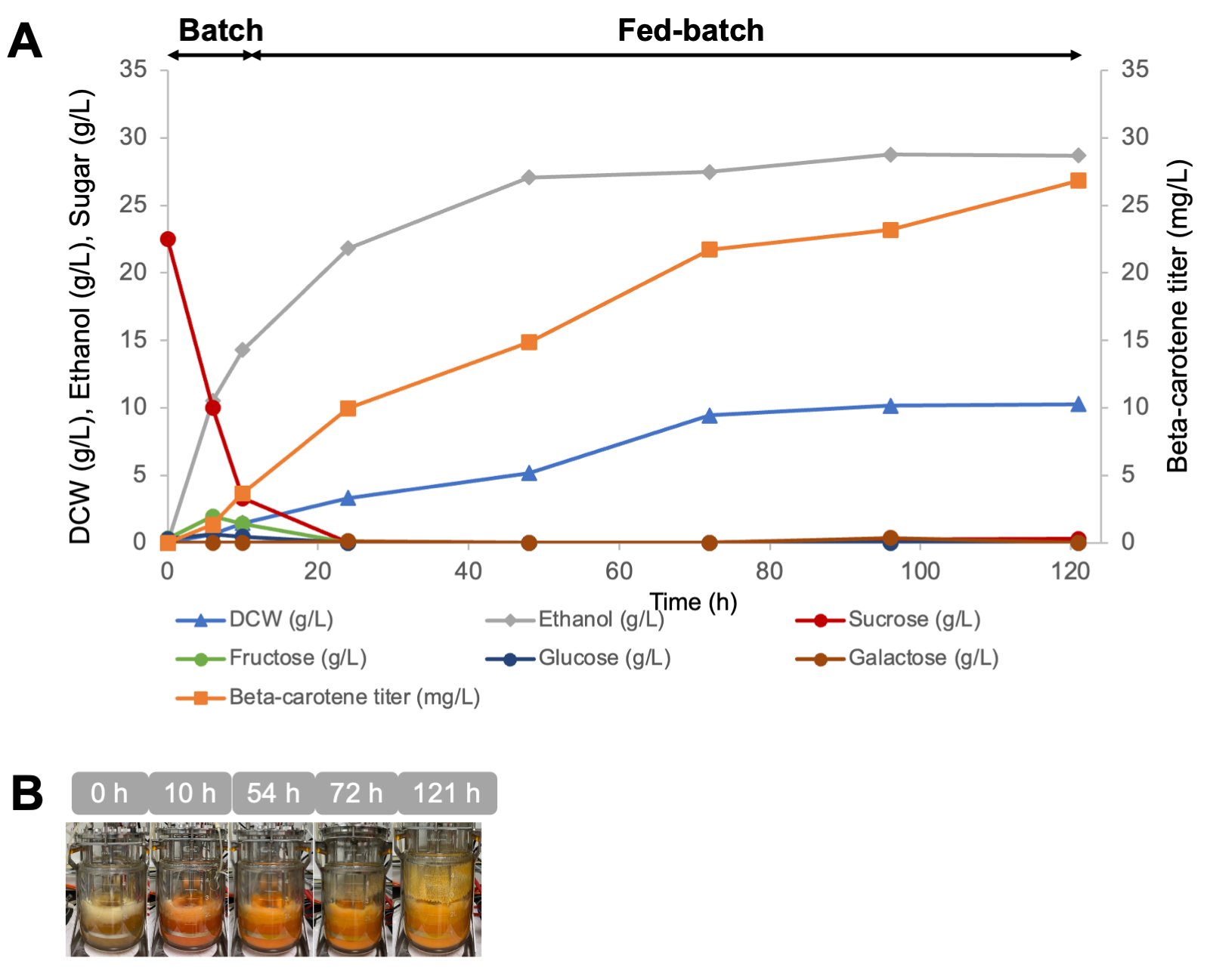
**

**Fig. S3 Fed-batch fermentation of Strain Sp_Bc ∆*gal80* in a 5-L fermenter using sucrose and galactose as mixed carbon sources.** (A) Fermentation profile including beta-carotene titer, biomass as dry cell weight, ethanol production, and residual sugars (sucrose, galactose, fructose, and glucose) over time. The fermentation began with a 2-L batch phase using a basal salt medium supplemented with 20 g/L sucrose. Subsequent fed-batch stages initiated at the 10th hour involved periodic additions of a solution of sucrose, galactose and yeast extract (at concentrations of 50, 50, and 15 g/L, respectively) at 5-hour intervals across 20 cycles, cumulatively reaching 120 g/L total sugars. (B) Visual progression of the bioreactor culture over time, with the intensifying orange hue reflecting the increasing beta-carotene concentration, a visual confirmation of successful carotenoid biosynthesis.

**
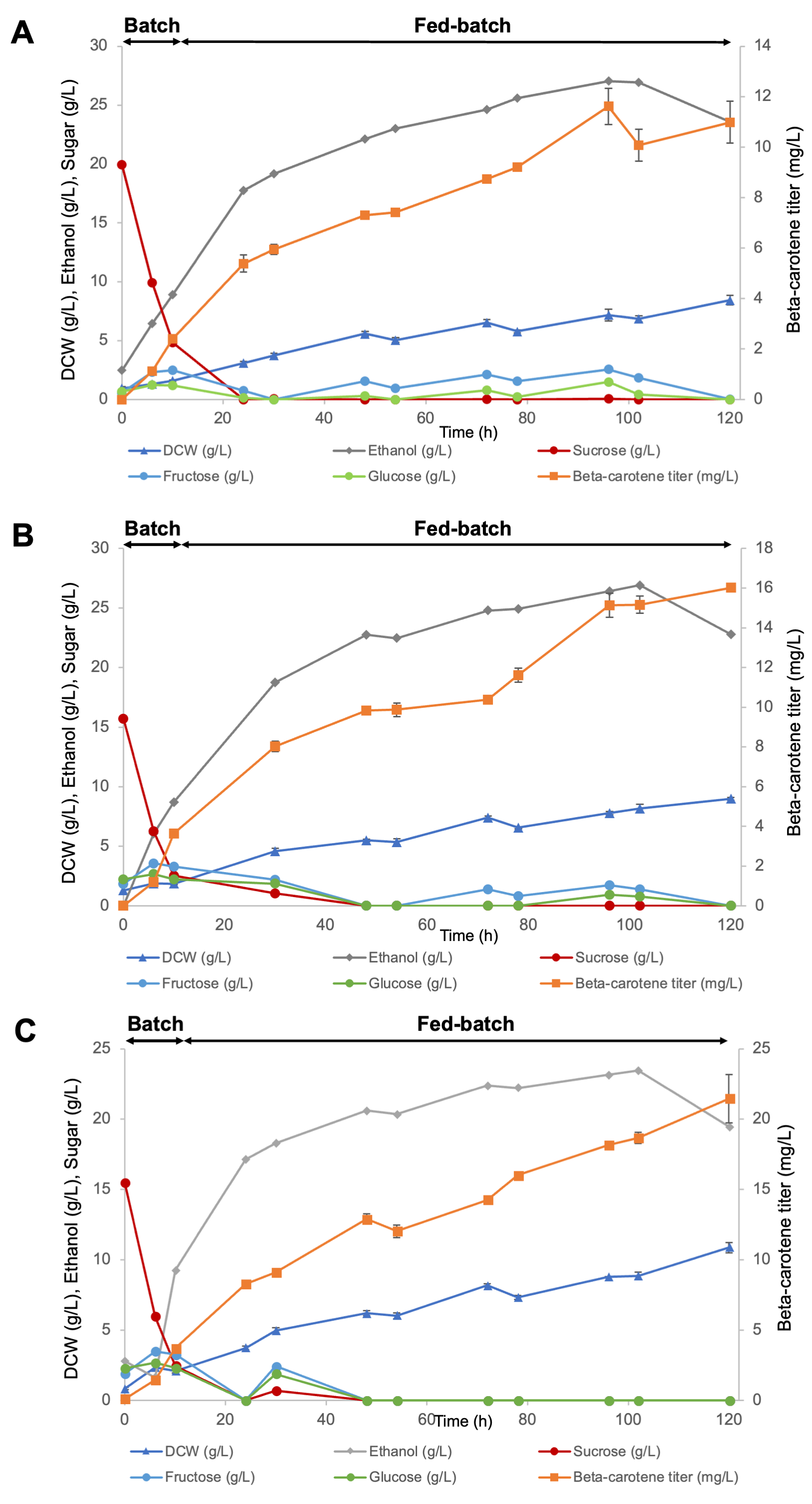
**

**Fig S4.** **Fed-batch fermentation of Strain Sp_Bc ∆*gal80* in a 5-L fermenter with fish meal as an alternative nitrogen source.**

The figures depict fermentation profiles including β-carotene titer, biomass as dry cell weight (DCW), ethanol production, and residual sugars (sucrose, fructose, and glucose), with averages and standard deviations from duplicate runs. Panel A shows 100% nitrogen substitution with 30 g/L fish meal, Panel B shows 90% substitution with 27 g/L fish meal, and Panel C shows 70% substitution with 21 g/L fish meal out of a total of 30 g/L nitrogen source. Error bars reflect standard deviations.
